# Centrosomes in the zebrafish (*Danio rerio*): a review including the related basal body

**DOI:** 10.1186/2046-2530-1-9

**Published:** 2012-06-07

**Authors:** Charles A Lessman

**Affiliations:** 1 Department of Biological Sciences, The University of Memphis, Life Sciences 223, Memphis, TN 38152, USA

**Keywords:** γ-tubulin, spindle, cilium, centriole, centrin, microtubule, MTOC

## Abstract

Ever since Edouard Van Beneden and Theodor Boveri first formally described the centrosome in the late 1800s, it has captivated cell biologists. The name clearly indicated its central importance to cell functioning, even to these early investigators. We now know of its role as a major microtubule-organizing center (MTOC) and of its dynamic roles in cell division, vesicle trafficking and for its relative, the basal body, ciliogenesis. While centrosomes are found in most animal cells, notably it is absent in most oocytes and higher plant cells. Nevertheless, it appears that critical components of the centrosome act as MTOCs in these cells as well. The zebrafish has emerged as an exciting and promising new model organism, primarily due to the pioneering efforts of George Streisinger to use zebrafish in genetic studies and due to Christiane Nusslein-Volhard, Wolfgang Driever and their teams of collaborators, who applied forward genetics to elicit a large number of mutant lines. The transparency and rapid external development of the embryo allow for experiments not easily done in other vertebrates. The ease of producing transgenic lines, often with the use of fluorescent reporters, and gene knockdowns with antisense morpholinos further contributes to the appeal of the model as an experimental system. The added advantage of high-throughput screening of small-molecule libraries, as well as the ease of mass rearing together with low cost, makes the zebrafish a true frontrunner as a model vertebrate organism. The zebrafish has a body plan shared by all vertebrates, including humans. This conservation of body plan provides added significance to the existing lines of zebrafish as human disease models and adds an impetus to the ongoing efforts to develop new models. In this review, the current state of knowledge about the centrosome in the zebrafish model is explored. Also, studies on the related basal body in zebrafish and their relationship to ciliogenesis are reviewed.

## Introduction

According to EB Wilson in his classic text, *The Cell in Development and Heredity *[1], Van Beneden first described the "polar corpuscle" in 1876 and Boveri later named it the "centrosome" in 1888. For more than a century, the centrosome has intrigued scientists and continues to do so today. Although much is now known about the centrosome, it remains somewhat mysterious, with many secrets left to reveal about its function and regulation. The zebrafish (*Danio rerio*), a small tropical freshwater teleost, has emerged as a model for cell and developmental biology because of its high fecundity, short generation time and rapid development of the externally fertilized and translucent embryos [2] (see also [3]). As a relatively new model organism, the zebrafish has attracted considerable attention in the scientific community due to its genetic tractability, speed of embryonic development and optical clarity. Many scientists espouse the hope that the advantages of the zebrafish model system will allow solutions to long-standing questions. For example, how is the centrosome regulated? Exactly what does it do in cell division? What is its relationship to basal bodies and ciliogenesis? It is the purpose of this review to summarize and outline the current state of knowledge about the centrosome and its relative, the basal body, in zebrafish.

Centrosomes in animal cells usually consist of γ-tubulin ring complexes (γ-TuRCs), centrioles, pericentriolar material and tubulins, along with a number of other centrosome-associated proteins. A previous proteomic analysis of isolated human centrosomes indicated about 70 protein components and revealed the complexity of the centrosome [4]. The major components are briefly reviewed regarding our knowledge of most animal cells and then those of the zebrafish in particular. Centrosomes and the related basal bodies of cilia are important microtubule (MT)-organizing centers (MTOCs) of animal cells. A number of serious diseases have been linked to their dysfunction (reviewed in [5]). A major centrosomal-basal body protein, γ-tubulin, a member of the tubulin superfamily, appears to play a central role in MTOC activity; nevertheless, our specific knowledge about γ-tubulin's role is far from complete. Like other tubulins, such as α- and β-tubulin, γ-tubulin exists as both a soluble and a polymer pool, albeit at about 1% the abundance of α- and β-tubulins. Very enlightening work on *Drosophila *and *Xenopus *has revealed that the γ-TuRCs are composed of about 13 γ-tubulin subunits, together with several different proteins called "γ-tubulin complex proteins" (γ-tubulin ring-associated protein, or GRIP) [6-8], and are found at the minus ends of microtubules associated with centrosomes. The association of the γ-TuRCs with the centrosome appears to be dynamic, as seen in *Caenorhabditis elegans *early cleavage embryos expressing γ-tubulin-GFP [9]. Clearly, the centrosome duplication and other dynamic processes are synchronized with the cell cycle, presumably by the cyclin-dependent kinase (CDK) system, but the exact mechanism by which they occur is unclear. How γ-tubulin and its associated proteins are recruited to and docked at the centrosome is also largely unknown. The microtubule cytoskeleton is essential for a variety of cellular processes, including cell movement, organelle transport and cell division and, for primary cilia, sensation and signaling. Moreover, in oocytes and early embryos, microtubules have been implicated in localization of important embryonic determinants such as *bicoid *mRNA in *Drosophila *[10] and *Vg1 *mRNA in *Xenopus *[11], as well as trafficking cell components such as β-catenin to cytokinesis furrows in cleaving zebrafish embryos [12]. Recently, it was reported that nocodazole, a MT inhibitor, abolished translocation of the dorsal determinant Wnt8a in zebrafish embryos, emphasizing the importance of MT in zebrafish development [13]. MTOCs organize MTs by initiating noncovalent assembly of αβ-tubulin heterodimers, anchoring them at their minus ends and facilitating MT extension at the rapidly growing plus ends. These complicated dynamic properties involve the functions of numerous MT-associated proteins. MTOCs thus organize and control the MT network both spatially and temporally, including that of cilia. They also help to define the MT surface lattice [14]. The morphology, subcellular localization and molecular make-up of MTOCs vary across different species and different cell types within single species.

Although the main topic of this review is the centrosome, it is becoming increasingly clear that centrosomes and basal bodies are intimately related, especially in some cell types, such as stem cells containing primary cilia. Basal bodies are derived from centrosome components, especially the mother centriole, which is thought to direct assembly of the axoneme (reviewed in [15-17]). During the cell cycle, the axoneme is disassembled and the basal body components are used to reconstitute the centrosome [18]. Thus the centrosome and basal body are intimately related and composed of many of the same components, and their formation is regulated in concert with the cell cycle.

## General centrosome structure and function

The centrosome and related basal body are thought to provide sensation, motion and cell division to the eukaryotic cell and thus are ancient structures believed to be found in the very first eukaryotes (reviewed in [19-21]). The centrosome and basal body structures are more than MTOCs [22] in that they may act as scaffolds for signal cascades and may contribute to disease states such as cancer [23] and ciliopathies [5]. In most animal cells, centrosomes are composed of a pair of centrioles surrounded by an amorphous cloud of electron-dense material, the pericentriolar material (PCM) [24]. Mass spectrometric analysis of purified human centrosomes has revealed 47 previously known centrosome components in four major protein groups: (1) structural components including tubulins, γ-tubulin complex components, centrins, A-kinase anchoring protein 450 (AKAP450), pericentrin, ninein, PCM-1 and centriole-associated proteins; (2) regulatory molecules, including cyclin/CDK1, protein kinase A (PKA), Plk1, type 1 pyrophosphatase (PPase 1) and PPase 2A; (3) motor proteins, including dynein and dynactins; and (4) heat shock proteins, including Hsp90 and Hsp73 [4,25]. An additional 64 proteins were newly identified. Many of these were coiled-coil domain proteins. The PCM is largely proteinaceous [26,27] and consists of a matrixlike structure (also referred to as the "centrosome scaffold") [24,28,29]. One characterized PCM protein required for *in vivo *MT nucleation is γ-tubulin. In *Drosophila *and *Xenopus*, γ-tubulin contributes to macromolecular complexes or γ-TuRCs, with a characteristic ring structure (approximately 25 nm in diameter), together with a variety of associated proteins [6,29-31]. Hundreds of 32S γ-TuRC tether to the centrosome scaffold to serve as the site of origin for MTs [24,28,29] and are required for spindle assembly and progression through mitosis [7,32-34]. Thus the centrosome is the primary site for MT nucleation, and the centrosomal γ-TuRC is thought to be necessary for anchoring of MT minus ends to centrosomes [35-39]. In addition to playing roles in centrosome-dependent MT nucleation [40], γ-tubulin has also been found to be closely associated with the PCM and centrioles and to be a core component of the centriole [41]. It plays a key role in daughter centriole formation [42] or in the nucleation and/or stabilization of the centriolar MTs [43].

## Pools of γ-tubulin

In mammalian cells, a significant fraction of the total pool of γ-tubulin is found in cytosolic complexes [43]. Inside the cell, γ-tubulin is located at the centrosome as γTuRCs and is also distributed in the cytoplasm as a soluble pool of inactive smaller complexes that constitute an exchangeable stock of material [43-46]. Recruitment of γ-tubulin to sperm basal bodies is necessary for the assembly of a MT nucleation-competent paternal centrosome [7,47]. Using immunofluorescence and GFP reporter construct techniques, γ-tubulin was shown to be associated with centrosomes dynamically in mitotic cells, being massively recruited at prophase and released at anaphase-telophase. This accumulation in mitotic centrosomes is dramatic during the first embryonic divisions in *C. elegans *[48]. Moreover, fluorescence measurements suggest that the amount of antigenic γ-tubulin increases during mitosis and that, in HeLa cells, the total amount of γ-tubulin in the spindle is larger than the amount of γ-tubulin in the spindle poles [49]. The cell-stage distribution of γ-tubulin varies between animal cells of different species and between cells of different tissues within the same species. These observations imply that both the localization and the concentration of γ-tubulin are highly regulated during the cell cycle and that the regulation may vary between cell types and between species [49]. Thus understanding how γ-TuRCs are recruited to and docked at the centrosome is essential for understanding the regulation of MT nucleating activity. The function of the soluble cytoplasmic γ-tubulin remains unclear. Also, very little is known about how the γ-TuRC is assembled and tethered in the centrosome. Moreover, the redistribution of centrosomal proteins to specific sites of the cell is poorly understood, and the mechanisms controlling MT nucleation within the living cell are still unclear. Thus, though considerable progress has been made in the understanding of centrosomes, significant work remains to be done. The zebrafish system shows promise in providing more insight.

## γ-Tubulin ring complex components in zebrafish

**Figure 1:**
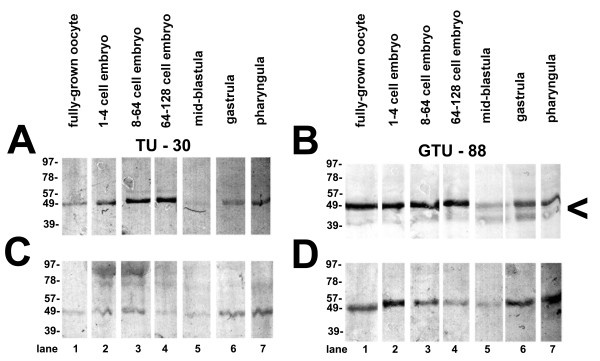
**Western blot of zebrafish oocyte or embryo extracts at the indicated developmental stages probed by anti-γ-tubulin antibodies (GTU-88 or TU-30)**. Molecular weight standards (in kilodaltons) are shown at left. Each sample lane was loaded with 0.8 mg of protein (wet weight equivalent). **(A) **Original 40,000 × *g *supernatant probed by TU-30. **(B) **Original 40,000 × *g *supernatant probed by GTU-88. Arrowhead indicates γ-tubulin breakdown product. **(C) **Original 40,000 × *g *pellet probed by TU-30. **(D) **Original 40,000 × *g *pellet probed by GTU-88. Lane 1: Fully grown oocyte. Lane 2: one to four cells. Lane 3: eight to sixty-four cells. Lane 4: 64 to 128 cells. Lane 5: Midblastula (> 1,000 cells). Lane 6: Gastrula. Lane 7: Pharyngula. Reprinted with permission [53].

**Figure 2:**
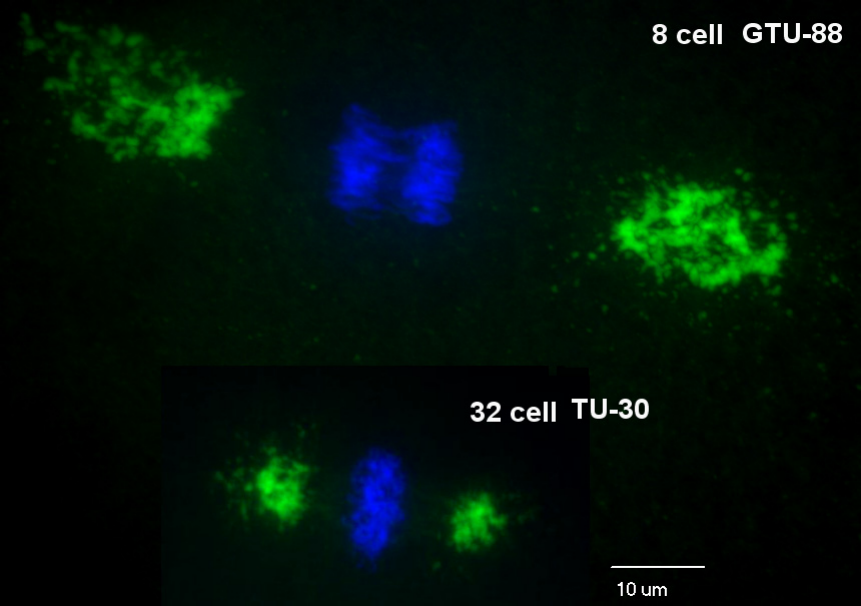
**Centrosomes at the spindle poles of the blastomere of an eight- or thirty-two-cell paraformalin-fixed zebrafish embryo depicted by γ-tubulin antibody GTU-88 or TU-30, respectively, and Alexa Fluor 488 dye-conjugated second antibody (green) as well as chromosomes stained with 4',6-diamidino-2-phenylindole (blue)**. *Z*-axis projections of approximately 60 optical sections photographed with an Olympus spinning disk confocal microscope using a 60 × oil immersion lens objective. Note the size differences, with the γ-tubulin array being smaller in centrosomes of 32-cell compared to 8-cell embryos.

In 1989, a new member of the tubulin family, γ-tubulin, was discovered in *Aspergillus nidulans *[32] and was found to be a major conserved component of the centrosome [33]. The discovery led to a major research effort over the next few years aimed at elucidating the structure and function of γ-tubulin. The primary protein sequence of γ-tubulin has been compared to α- and β-tubulins [50,51], as well as to other tubulin family members, including δ- and ε-tubulins [52]. These results indicated a high degree of sequence homology when comparisons among divergent species were made. For example, α-tubulins, β-tubulins and γ-tubulins were, respectively, 89% to 95%, 88% to 94% and 72% to 94% homologous. The δ- and ε-tubulin family members were the most varied, having 47% to 57% and 58% homology, respectively. Such high degrees of homology may allow the use of antibodies developed for one species in another species. This is the case for γ-tubulin in zebrafish. The mouse monoclonal antibodies GTU-88 and TU-30 were demonstrated to cross-react with zebrafish γ-tubulin on Western blots (Figure 1) [53]. Both antibodies label centrosome spindle poles in zebrafish early embryos as well (Figure 2). For example, GTU-88 recognizes the fully conserved epitope 38 EEFATEGTDRKDVFFY 53 in the N terminus of human and zebrafish γ-tubulin. TU-30 recognizes the epitope 434 EYSAATRPDYISWGTQ 449 at the zebrafish γ-tubulin C terminus. Human γ-tubulin (the antigen sequence used) has one amino acid difference in the TU-30 epitope, with H at residue 436 instead of S in zebrafish. Armed with these antibodies, investigators, including those working on zebrafish centrosome questions could make progress in understanding how centrosomes function. For example, large accumulations of γ-tubulin have been found to be associated with spindle poles of early cleavage stages [54].

**Figure 3:**
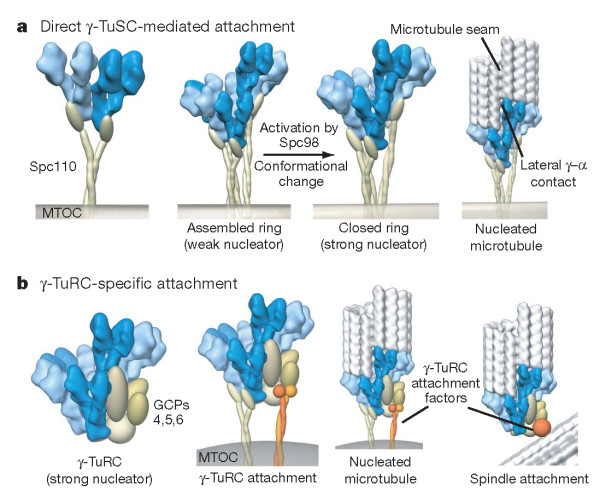
**Models of nucleation complex attachment and activation**. **(a) **In the absence of c-tubulin ring complex (c-TuRC)-specific components, as in *Saccharomyces*, Spc110 or its equivalent directly attaches c-tubulin small complex (c-TuSC) to microtubule-organizing centers (MTOCs), promoting ring assembly. **(b) **In organisms with complete c-TuRCs, active complexes attach to organizing centers directly through c-TuSCs or potentially through unique sites in the c-TuRC-specific components. Localization of c-TuRCs at non-MTOC locations, such as within the mitotic spindle, is mediated through the c-TuRC-specific proteins. In both models, c-TuSC interactions define the geometry of the nucleating template. Reprinted with permission [59].

Although it was previously known that αβ-tubulin dimers assemble *in vitro *under certain conditions, it was also clear that, for animal cells, centrosomes are required for *in vivo *MT assembly [28]. Then investigators found evidence of large complexes containing γ-tubulin associated with the minus ends of MTs at the centrosome [7]. However, γ-tubulin was also found to be associated with the mitotic spindle [49] and the midbody at cytokinesis [55]. Groups led by Bruce Alberts simultaneously reported in the journal *Nature *the structure of *Xenopus *γ-tubulin ring complexes associated with the centrosome and hypothesized that these acted as templates for nucleating new MTs [8,29]. The complexes (later termed "γ-TuRCs") were found to contain at least seven distinct proteins and to have an open ring structure with a diameter of 25 nm [31]. Centrosomes can be disassembled by high salt to reveal a filamentous "centromatrix" onto which γ-TuRCs can be reassembled *in vitro *[56]. In addition, *Drosophila *embryos have two distinct γ-TuRCs, large 2.2 MDa and small 280 kDa (γ-TuSC) versions with differing MT nucleating capabilities [6,57,58]. The smaller version is thought to be the essential core consisting of two γ-tubulin subunits and one copy each of spindle pole body components (Spc), Spc97 and Spc98 [59], with the latter being yeast homologs [60] of γ-tubulin complex proteins 2 and 3, respectively [61]. The γ-TuSCs form filaments with 13-part symmetry similar to a MT and have been modeled to nucleate MTs in various systems (Figure 3) [59,60].

**Figure 4:**
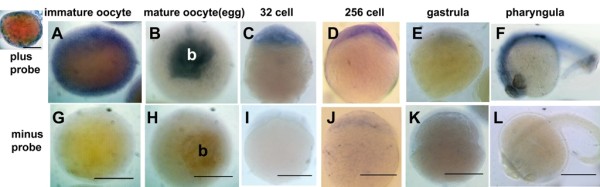
***In situ *hybridization (probed with fluoroscein isothiocyanate-oligo-γ-tubulin probe) of different stages of zebrafish oocytes and embryos**. **(A) **and **(G) **Fully grown, immature oocytes. **(B) **and **(H) **Mature oocytes or eggs) and embryos. **(C) **and **(I) **32-cell embryos. **(D) **and **(J) **256-cell embryos. **(E) **and **(K) **Approximately 30% epiboly gastrula. **(F) **and **(L) **pharyngula). The top panels in (A) through (F) show the "with probe" treatments, and the lower panels in (G) through (L) show the corresponding "without probe" treatments. The primordial blastodisc (b) that forms at the animal pole of the egg during oocyte meiotic maturation labeled intensely with the probe (B) Inset: Immature oocyte probed after hemisection showing cortical label. Anti-FITC secondary antibody conjugated to alkaline phosphatase and the substrate 3,3'-diaminobenzidine were used to develop color. The specimens were dehydrated in 100% MeOH and placed in clearing media containing benzyl benzoate and benzyl alcohol (2:1 dilution) prior to being mounted on slides. Scale bar = 250 μm. Reprinted with permission [64].

Zebrafish oocytes and embryos have large complexes containing γ-tubulin that range in size to over 2 MDa by column chromatography [53]. These results are similar to those found in *Xenopus *and *Drosophila *[31] and suggest that a large pool of γ-TuRCs is stockpiled in the oocyte for use in the embryo, especially during the very rapid divisions of cleavage. Once cleavage begins in the zebrafish embryo, cell division occurs astonishingly fast, every 15 to 20 minutes [62,63]; thus, within 3 hours postfertilization, the embryo has over 1,000 cells, each of which has a centrosome! After reaching the midblastula transition, cell divisions slow somewhat, the maternal reserves of protein and mRNA decrease and the zygotic genome is first significantly expressed [63]. This applies also to γ-tubulin protein (Figure 1) and mRNA (Figure 4) in zebrafish embryos as both decrease during midblastula to early gastrulation and return to higher levels subsequently [53,64].

A National Center for Biotechnology Information database search revealed γ-tubulin complex proteins 2 to 5, HAUS augmin-like protein 6 and mitotic spindle-organizing proteins 1 and 2 in the zebrafish (Table 1). Generally, the γ-tubulin complex protein 2 through 5 sequence homology was high compared with other vertebrates. This finding is in agreement with a report of a cross-reaction of zebrafish proteins with *Xenopus *Xgrip 109 (analogous to Spc98 and γ-complex protein 3 (GCP3)) and Xgrip 195 polyclonal antibodies [53]. A putative zebrafish ortholog for a newly discovered human γ-TuRC component, GCP8, has recently been reported [65]. GCP8 is a small protein (about 20 KDa) that may be part of the core γ-TuSC.

**Table 1:** Results of an NCBI database search for γ-tubulin complex component proteins in zebrafish^a^

Protein	Amino acids	Cal. MW (Da)	Identity (%)	Gap (%)
γ-tubulin complex protein 2	882	101,642	79 to 88	0
γ-tubulin complex protein 3	899	102,403	77 to 79	3
γ-tubulin complex protein 4	668	76,008	88 to 89	0
γ-tubulin complex protein 5	1015	117,437	72 to 73	1 to 2
HAUS augmin-like protein 6	794	89,594	30 to 48	5 to 8
Mitotic spindle-organizing protein 1	75	8,051	80 to 90	0
Mitotic spindle-organizing protein 2	153	16,177	52 to 61	5 to 8

^a^NCBI, National Center for Biotechnology Information. The molecular weight (MW) was calculated (Cal.) on the basis of the primary sequence using the program available at http://www.encorbio.com/protocols/Prot-MW.htm (EnCor Biotechnology Inc, Gainesville, FL, USA). The identity and gap percentages are based on blastp of zebrafish protein sequences with other vertebrates, including human and mouse.

## General centriole structure and function

Centrioles are cylindrical structures with ninefold radial symmetry and triplets of short MTs forming the outside wall in many species. A pair of centrioles oriented to each other at about a right angle is found in centrosomes and basal bodies [66]. Although centrioles are found in most animal cells, they are notably absent in oocytes and eggs until the latter are fertilized. The sperm provides the initial centriole to reconstitute centrosomes in the zygote [67]. In preparation for cell division, a procentriole, or daughter, forms at the base of each mother centriole by first forming a cartwheel structure, then forming MTs [66]. The cartwheel has a ninefold symmetry composed of spindle assembly abnormal SAS-6 homodimers with coiled-coil domains radiating outermost as spokes [68]. This process of centriole replication or procentriole formation occurs in two ways: (1) the canonical pathway, involving mother-daughter proximity and resulting in exactly one net new centriole pair and (2) the *de novo *pathway, particularly active in ciliogenesis in ciliated vertebrate epithelial cells, involving multiple procentriole formations from fibrous granules and deuterostomes not necessarily containing existing centrioles [66]. The mother centriole is older and has specializations that include filaments or appendages [66] and additional proteins such as centriolin [69]. In addition, the mother centriole produces the cilium from the basal body [66]. Questions remain about the underlying factors that produce differences between the mother and daughter centrioles' ability to organize centrosomes or basal bodies and, in turn, to organize a spindle or a cilium, respectively. Other areas of centriole and/or basal body biology that need further investigation include (1) temporal and genetic factors involved in aging and positioning of centrioles relative to the formation of centrosomes versus basal bodies, (2) the role of centriole condition in stem cell maintenance or differentiation and (3) centriole fate and mechanism of loss in oocytes and other cells that lose centrioles normally (reviewed in [70]).

## The centriole in zebrafish

**Figure 5:**
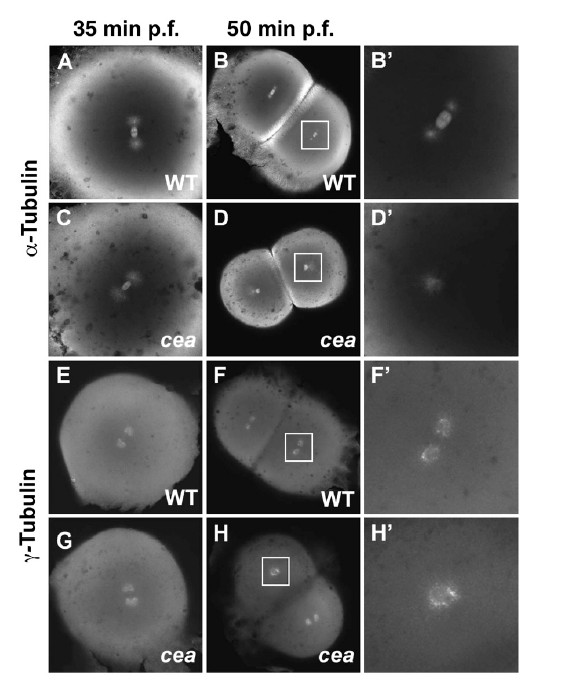
**Spindle organization and centrosome duplication defects in maternally mutant cellular atoll (*cea*) zebrafish embryos**. **(A) **to **(D') **Embryos labeled with anti-α-tubulin antibody. (A) and (B') Fixed wild-type embryos. (C) and (D') Mutant embryos. Spindle organization is normal in the mutant embryos immediately prior to the first cell division (35 minutes postfertilization (pf) (C); compare with (A)) but is defective in a fraction of blastomeres in the next cleavage cycle (50 minutes pf (D) and (D'); compare with (B) and (B')). (E) to (H') Embryos labeled with anti-γ-tubulin antibody. (E) and (F') Fixed wild-type. (G) and (H') Mutant. Centrosome duplication appears normal immediately prior to the first cleavage division (35 minutes pf (G); compare with (E)) but is defective in a fraction of the blastomeres in the following cleavage cycle (50 minutes pf (H) and (H'); compare with (F) and (F')). Animal views: (B'), (D'), (F') and (H') are enlargements of the area indicated by the squares in (B), (D), (F) and (H), respectively. Reprinted with permission [71].

**Figure 6:**
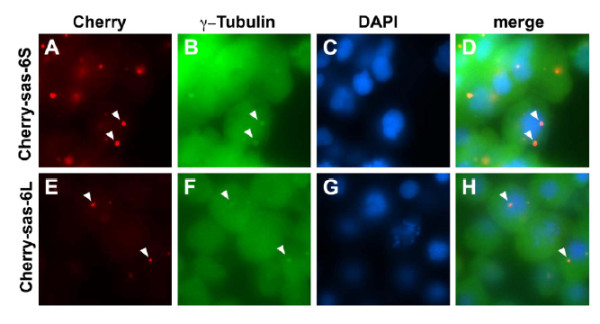
**Cea/Sas-6 localizes to zebrafish centrosomes**. Expressed fusions of mCherry and Sas-6 splice forms colocalize in cytoplasmic structures containing γ-tubulin (arrowheads), markers for centrosomes. Fields of cells in embryos are fixed at 50% epiboly. Reprinted with permission [71].

**Figure 7:**
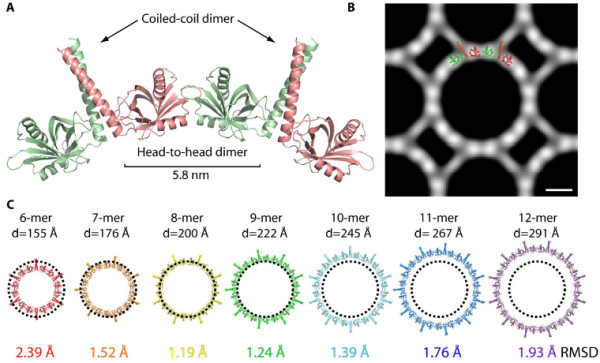
**Model of SAS-6 ring assembly in zebrafish**. **(A) **Ribbon presentation of a modeled SAS-6 tetramer based on the observed coiled-coil and head-to-head dimers. The distance between the base regions of the two coiled-coil domains is indicated. **(B) **Cryo-electron microscopic image of a face-on view of a thin crystal of N-SAS-61-217. The pixel size is 3.74 Å/pixel. Scale bar = 60 Å. The image was Fourier-filtered and symmetry-averaged. Overlay shows the SAS-6 tetramer presented in (A). The overlaid structure is based on N-SAS-61-179, which has a shorter coiled-coil domain than N-SAS-61-217. **(C) **Models of SAS-6 rings with different symmetries. The approximate diameters of these rings (the double-distance from head domain center to ring center) are indicated above the modeled rings. The diameter of cartwheel hubs observed in procentrioles by cryo-electron tomography is shown as a dotted circle. To model these rings, a change in the orientation of the head domains relative to the coiled-coil domain was allowed for. To compare the required changes, the root-mean-square deviation between the N-SAS-61-179 structure and two equivalent head domains in the modeled ring was calculated. These values are indicated under the modeled rings. Reprinted with permission [74].

A female sterile maternal effect mutation in the *cellular atoll *(*cea*) gene in zebrafish produces aberrant mitosis beginning with the second cleavage [71,72]. Maternally mutant *cea *embryos produce normal mitotic spindles for the first cleavage only and thereafter fail to produce normal spindles (Figure 5). Interestingly, paternal *cea *mutants mated with wild-type females produce defects in the first cleavage spindle, but normal ones subsequently. C*ea *encodes SAS-6, a coiled-coil protein associated with the centrioles and SAS-6 splice variants fused to mCherry, colocalize with γ-tubulin at the centrosomes (Figure 6). Very recently, it was reported that, in cultured cells, SAS-6 is targeted by an E3-ubiquitin ligase that uses an F-box protein, FBXW5, as a targeting subunit [73]. FBXW5 is in turn regulated by Polo-like kinase 4 (PLK4), thus degradation of SAS-6 by this mechanism limits centrosome reduplication. It will be interesting to see if FBXW5 acts in a similar way in zebrafish. SAS-6 has been shown to have a nine-part "cartwheel" oligomeric structure, suggesting a template function for centriole assembly in *C. elegans *[68] and, importantly, for zebrafish as well [74]. The homodimers of SAS-6 with ninefold symmetry were shown to fit most closely with actual procentriole cartwheels compared with those with other symmetries (Figure 7) [74]. Work in *Drosophila *has also implicated PLK4 in centriole assembly, but in this system it pairs with SAS-4 and *asterless *(*Asl*) (CEP152 in humans) [75].

**Figure 8:**
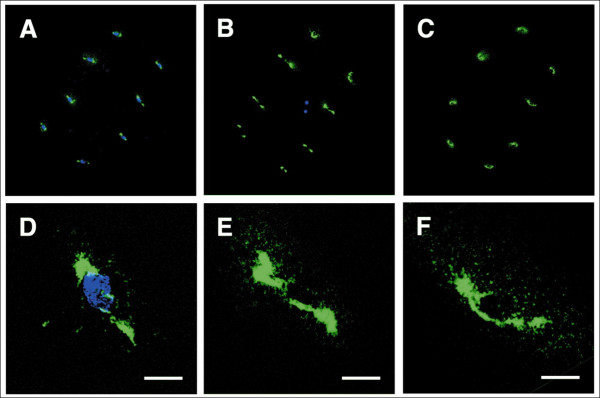
**Centrosome duplication is not affected in futile cycle (*fue*) mutants**. Centrosomes at the eight-cell stage during interphase visualized with an antibody directed against γ-tubulin (green) and nuclei labeled with 4',6-diamidino-2-phenylindole (blue) from **(A) **a wild-type embryo, **(B) **a *fue *embryo and **(C) **an embryo treated with the DNA replication inhibitor aphidicolin. **(D) **through **(F) **Enlargements of single-centrosome pairs shown in (A) through (C), respectively. Centrosomes are present and able to duplicate in all *fue *cells as well as the (anucleate) cells of aphidicolin-treated embryos, demonstrating that the centrosomes can duplicate independently of nuclei. Scale bars = 20 mm. Reprinted with permission [54].

The maternal effect gene *futile cycle *(*fue*) is necessary for pronuclear congression and for normal mitotic spindle formation [54] in cleaving zebrafish embryos. It is particularly notable that *fue *mutant embryos seem to produce normal centrosomes (Figure 8) even when DNA replication and mitotic spindle assembly are deranged. This may be due to a reduced level of cell-cycle checkpoint surveillance in cleaving embryos [76]. Cytokinesis furrow formation is impaired in the mutant phenotype of another zebrafish maternal effect gene called *cellular island *(*cei*), but mitotic spindle formation appears normal [77]. Cleaving embryos produce a transient furrow MT array (FMA) that consists of MTs oriented perpendicular to the cleavage furrow [77]. The FMA is thought to help traffic cytoskeletal components such as β-catenin to the furrow [12]. Thus the *cei *mutant phenotype indicates that the FMA and spindle MTOCs must be separate because spindles are normal, but FMAs are not. The idea of multiple MTOCs in a given cell is not novel, as there are many examples described in the literature, such as ciliated epithelial cells that may have many basal bodies (reviewed in [78]).

## Centrins

Centrins are small Ca^2+^-binding proteins of the EF-hand superfamily related to calmodulin, troponin C and parvalbumin [79] that are associated with centrioles, but also may be found elsewhere, such as in the nucleus. In human cells, more than 90% of the centrin is not centrosome-associated [80]. In mammalian cells, centrin 2 is required for centriole duplication [81], and use of GFP-centrin constructs have shown differential behavior of mother and daughter centrioles [82,83]. Some centrins are usually associated with centrosomes, except in preimplantation porcine embryos [84], and are also found in basal bodies, including the connecting cilium of photoreceptors [79]. Centrin 2 has been shown to self-assemble into fibers in the presence of Ca^2+ ^reminiscent of those associated with basal bodies [85]. In addition, a *Chlamydomonas *centrin mutant has acentriolar spindle poles and lacks rhizoplasts that normally join the centrioles to the nucleus and result in increased chromosome loss and genomic instability [86]. Phosphorylation of human centrin at serine 170 during the G_2_/M phase has been implicated as the signal for centriole separation before centrosome duplication [87]. Another prominent signaling role for centrin is its interaction with transducin within the connecting cilium of photoreceptor cells [88].

## Centrin in zebrafish

In zebrafish, centrin constructs containing reporters have been used to indicate centrosome position in migrating neurons *in vivo *[89], polarization of retinal ganglion cells [90] and interkinetic nuclear migration in retinal cells [91]. A transgenic mouse line expressing GFP-centrin 2 driven by a constitutive promoter was found to display a single pair of fluorescent centrioles in most cells examined; however, it was noted that some cells of the adult brain seemed to accumulate aggregations of fusion protein [92]. Overexpression of GFP-centrin was found to produce large aggregates that caused disarray of the wild-type centrin in *Chlamydomonas *[93]. Thus caution should be exercised when utilizing centrin reporter constructs. A comparison of zebrafish, mouse and *Xenopus *centrin 2 protein sequences reveals a very high degree of homology, suggesting that reagents, such as antibodies, developed for mammals may also be useful in zebrafish (Table 2).

**Table 2:** Centrin 2 amino acid sequence comparison among zebrafish, *Xenopus *and mouse^a^


1 MASNYKKPSLGVTTQRKKPVPKPELTEEQKQEIREAFDLFDTDGAGTIDVKELKVAMRAL 60 XENTR
1 MASNFKKTTMASSAQRKRMSPKPELTEDQKQEIREAFDLFDADGTGTIDIKELKVAMRAL 60 MOUSE
1 MASGFRK-SSASANQRKKAGPKPELTEEQKQEIKEAFDLFDTDGSGTIDVKELKVAMRAL 59 DANRE

61 GFEPKKEEIKKMIADIDKEGTGKISFGDFMSAMTQKMAEKDSKEEIMKAFRLFDDDETGK 120 XENTR
61 GFEPKKEEIKKMISEIDKEGTGKMNFSDFLTVMTQKMSEKDTKEEILKAFKLFDDDETGK 120 MOUSE
60 GFEPKKEEIKKMIADIDKEGSGVIGFSDFLSMMTQKMSEKDSKEEILKAFRLFDDDCTGK 119 DANRE

121 ISFKNLKRVAKELGENLTDEELQEMIDEADRDGDGEVNEQEFLRIMKKTSLY- 172 XENTR
121 ISFKNLKRVAKELGENLTDEELQEMIDEADRDGDGEVNEQEFLRIMKKTSLY- 172 MOUSE
120 ISFKNLKRVAKELGENLTDEELQEMIDEADRDGDGEINEQEFLRIMKKTNLYG 172 DANRE

^a^*Xenopus *centrin 2 (Q28HC5_XENTR), mouse centrin 2 (CETN2_MOUSE) and zebrafish centrin 2 (B1P0R9_DANRE). Numbers refer to primary structure.

## Pericentrin

Antibodies from human scleroderma patients have been used to discover another centrosomal protein, called "pericentrin," with a molecular weight of about 220 kDa [36]. Pericentrin was found to associate with γ-TuRCs and form a lattice that enlarges, then disassembles with the cell cycle [35]. Kendrin, a larger splice variant (about 380 kDa) of the *PCNT *pericentrin gene, was discovered in human cells and was found to bind calmodulin [94,95]. In kendrin, also called "pericentrin B," overexpression was found in carcinoma cells known to have centrosomes of abnormal size and number [94]. Some uncertainty exists regarding the number and size of pericentrin isoforms and splice variants [96]. Nevertheless, pericentrin derangement has been implicated in a number of disease states, including Seckel syndrome, microcephalic osteodysplastic primordial dwarfism, cancer, ciliopathies and mental disorders such as schizophrenia [97]. It has been suggested that this diversity of diseases is attributable to the many binding partners of pericentrin, including γ-tubulin, γ-TuRCs, PCM1, AKAP450, DISC-1, Chk1, kinases, intraflagellar transport (IFT) and PC2 [97]. Pericentrin and AKAP450 bind to a γ-tubulin complex binding protein called CDK5RAP2 via a CM-2-like motif that is also conserved in zebrafish [98].

## Basal bodies in zebrafish and relation to cilia

**Figure 9:**
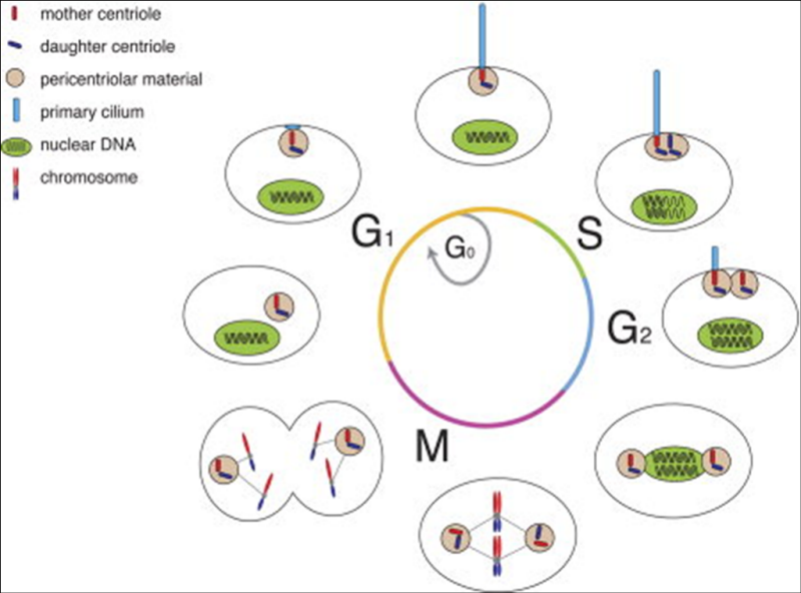
**Dual use of the centrioles during cell cycle and primary cilium formation**. In most cells, primary cilium formation first occurs during the G_1 _phase following centrosomal docking to the membrane. Intraflagellar transport (IFT) and accessory proteins build the ciliary axoneme, which extends directly from the mother centriole's triplet microtubules. During this stage of the cell cycle, as well as during the G_0 _phase, the cilium functions as a cellular antenna, interpreting extracellular signals such as Hedgehog and platelet-derived growth factor (PDGF). Upon entry into the S phase, the cell's centrioles and the DNA begin to replicate. The centrioles reach maturity during the late G_2 _phase, at which point the cilium is disassembled so that the engaged centrioles can be liberated for mitotic spindle formation. Once cell division is complete, the centrioles can proceed to ciliary reassembly in G_1_. Reprinted with permission [18].

Ciliogenesis and the cell cycle are interrelated, as are the basal body and centrosome (Figure 9). This relationship has garnered considerable scientific interest recently [16,17,99-102]. On the basis of this work, cilia, especially primary cilia, have assumed a new elevated status since the discovery of important functions provided by these organelles. These functions include (1) extracellular signaling for Sonic hedgehog, *wnt *and platelet-derived growth factor pathways; (2) sensing fluid flow in kidney tubules and cerebrospinal ventricles; and (3) determination of bilateral left-right patterning. These functions are in addition to the well-known cellular functions related to cilia, including cell motility, directed fluid flow and sensory transduction, including vision, hearing and equilibrium. Recent work indicates the importance of primary cilia to disease states such as cancer, and "ciliopathies" such as retinal degeneration, polydactyly, *situs inversus*, mental retardation, encephalocele and cysts in the kidney, liver and pancreas [17]. Organizing the primary cilium is the γ-tubulin-containing basal body that is derived from the mother centriole of the centrosome [99]. The basal body and the primary cilium are assembled and disassembled in tune with the cell cycle by an unknown mechanism [17]. A particularly important finding is the discovery that critical signaling pathways are concentrated in the primary cilium, including Sonic hedgehog-Patched and *Wnt*-Frizzled ligand receptor systems [17]. Primary cilia are easily overlooked because many are rather short, about 5 μm or less in length, and immotile, and only one per cell is usually present [103]. Markers that may be used for primary cilia include acetylated α-tubulin (axoneme), γ-tubulin (basal body) and the Patched and Frizzled receptors (cilium membrane). The cilium is produced from the basal body, which in turn is derived from the centrosome [15,104]. Other factors that promote extension and maintenance of the cilium include the IFT components including IFT-A, IFT-B, dynein 2 and kinesin 2.

Zebrafish primary cilia have been described or alluded to in an expanding number of recent reports [105-126]. A particularly informative study using the maternal-zygotic *oval *(MZ*ovl*; *ift88*) zebrafish mutant that lacks all cilia revealed dampened Hedgehog signaling but normal Wnt signaling [108]. The MZ*ovl *mutants had normal basal bodies but failed to localize Smoothened to the cell membrane in association with basal bodies. In addition, left-right patterning was deranged, and the mutants also developed pronephric cysts and pericardial edema indicative of ciliopathies. Thus, though basal bodies are required, they do not appear to be sufficient for ciliogenesis or cilia maintenance. Other gene products reported to be involved in ciliogenesis in zebrafish include the zinc-finger protein iguana [118], geminin [119], fused [123], fibroblast growth factors FGF8 and FGF24 [115], Smoothened [106], the *fleer *gene product [112], cdc14B phosphatase [109], Cep70 and Cep131 [122], Rab11, Rabin8 and transport protein particle II (TRAPPII) [121] and Nde-1 [110].

## Zebrafish photoreceptors and basal bodies

Gene knockdown of the centrosomal protein Cep290 resulted in zebrafish with visual defects and other symptoms of ciliopathies similar to those seen in Leber's congenital amaurosis, Meckel-Gruber syndrome, Joubert syndrome, Senor-Loken syndrome and Bardet-Bledl syndrome [116]. In addition, the visual defect may be rescued by expressing the N-terminal region of the human Cep290 protein in the zebrafish. Cep290 is one of eleven ciliopathy genes that include cc2d2a and involve retinal dystrophy as well as other defects, such as polycystic kidney [117]. The cc2d2a protein localizes to the photoreceptor-connecting cilium transition zone, and mutations result in visual defects as determined on the basis of electroretinograms in zebrafish [117].

## Conclusions

**Figure 10:**
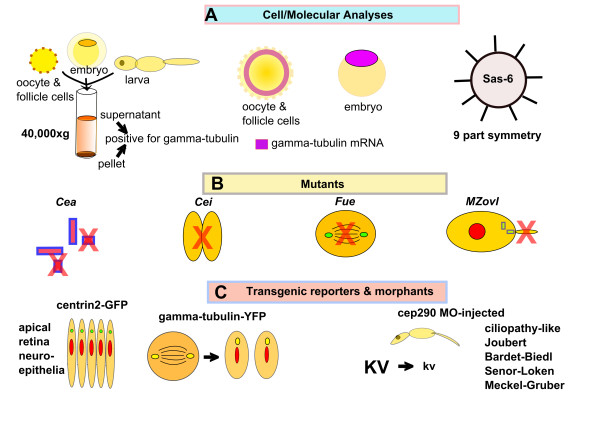
**Summary of centrosome and basal body research in zebrafish**. **(A) **Cellular and molecular findings include, from left, (1) Western blot with anti-γ-tubulin demonstrating γ-tubulin in 40,000 × *g *supernatants and pellets of ovarian oocytes and different embryonic stages [53], (2) *in situ *hybridization of γ-tubulin probe to mRNA in the cortices of ovarian oocytes and in embryonic cells [64] and (3) ninefold homodimer of coiled-coil sas-6 protein reveals structural basis for centriole assembly in zebrafish [74]. **(B) **The results of studies of mutants include, from left, (1) failure to replicate centrioles due to sas-6 mutation in *cellular atoll *(*cea*) [71], (2) failure to organize a furrow microtubule array due to aurora B kinase mutation in *cellular island *(*cei*) [77], (3) failure to organize DNA and spindle, though centrosome duplication and placement are normal in *futile cycle *mutant (*fue*) [54] and (4) the maternal-zygotic *ovl *(MZ*ovl*) mutant lacks cilia due to *IFT88 *mutation, though basal bodies appear normal [108]. **(C) **The results of transgenic reporters and morphants in zebrafish, from left, (1) centrin 2-GFP marks apical polarity in retinal neuroepithelium development, as does γ-tubulin-YFP [91], and (2) Cep290 morpholino injection produces morphants with curved body phenotype and reduced Kupffer vesicle size characteristic of mutations in ciliopathy-associated genes; analogous gene mutations cause a number of human ciliopathy syndromes [116].

The zebrafish model system shows promise in providing answers to long-standing questions involving the functions of centrosomes and the related basal bodies. This seems true in view of the genetic tractability of the zebrafish, that is, the many mutant and transgenic lines available, its favorable optical properties, ready accessibility to all stages of the life cycle and the possibility of high-throughput screening for therapeutic agents [127]. The major findings of centrosome and/or basal body research in zebrafish are summarized in Figure 10. Both γ-tubulin and γ-TuRC components, as well as SAS-6, the "cartwheel" centriole template, have been found in zebrafish. Zebrafish mutants such as *cea*, *cei *and *fue *have shown promise in providing insights into centrosome function. Centrin transgenic reporters in zebrafish have been useful in tracking centrosome position *in vivo*. Furthermore, zebrafish mutants such as MZ*ovl*, which lack cilia, may prove critical to understanding basal body-cilia dynamics and photoreceptor biology. Thus the zebrafish is proving to be a useful model with which to complement other vertebrate systems, such as the mouse, allowing experiments on and manipulations of developing embryos that are difficult or not feasible in mammals.

## Abbreviations

AKAP: A-kinase anchoring protein; *Asl*: Asterless; cdc: cell division control; CDK5RAP2: cyclin-dependent kinase 5 regulatory subunit-associated protein 2; *cea*: cellular atoll; *cei*: cellular island; Cep: centrosomal protein; ChK-1: checkpoint 1 kinase; CM2: centrosomin 2; DAPI: 4',6-diamidino-2-phenylindole; DISC-1: disrupted in schizophrenia 1; FGF: fibroblast growth factor; FITC: fluoroscein isothiocyanate; FMA: furrow microtubule array; *fue*: futile cycle; GCP: γ-complex protein; GFP: green fluorescent protein; GRIP: γ-tubulin ring-associated protein; γ-TuRC: γ-tubulin ring complex; γ-TuSC: γ-tubulin small complex; Hsp: heat shock protein; hpf: hours postfertilization; IFT: intraflagellar transport; kDa: kilodalton; MDa: megadalton; MT: microtubule; MTOC: microtubule-organizing center; MZ*ovl*: maternal-zygotic oval; Nde: nuclear distribution protein; PC2: polycystin; PCM: pericentriolar material; PDGF: platelet-derived growth factor; Plk: polo kinase; PKA: protein kinase A; PPase: protein phosphatase; SAS: spindle assembly abnormal; SPC: spindle pole body component; TRAPPII: transport protein particle II; wnt: Wingless integration; YFP: yellow fluorescent protein.

## Competing interests

The author declares that he has no competing interests.

## Authors' contributions

CAL prepared the confocal microscopy specimens and carried out the literature research, sequence alignment and manuscript preparation.
